# Angioedema Activity Score (AAS): A Valid and Reliable Tool to Use in Asian Patients

**DOI:** 10.1155/2019/9157895

**Published:** 2019-10-31

**Authors:** Kanokvalai Kulthanan, Leena Chularojanamontri, Chuda Rujitharanawong, Puncharas Weerasubpong, Karsten Weller, Marcus Maurer

**Affiliations:** ^1^Department of Dermatology, Faculty of Medicine, Siriraj Hospital, Mahidol University, Bangkok 10700, Thailand; ^2^Dermatological Allergology, Allergie-Centrum-Charité, Department of Dermatology and Allergy, Charité–Universitätsmedizin Berlin, Berlin, Germany

## Abstract

The Angioedema Activity Score (AAS) is recommended by the EAACI/GA^2^LEN/EDF/WAO guidelines for urticaria as the standard measure for assessing disease activity in patients with recurrent angioedema (RAE). To date, it has been translated into 80 languages for use in 52 countries, but it has not been formally validated in Asian patient populations. As RAE may be different in Asian and non-Asian patients, it is important to validate and characterize the reliability of tools to assess RAE disease activity in Asian patients. This study proposed to demonstrate the validity and reliability of the AAS in Asian patients. Accordingly, this study aimed to generate and validate the Thai version of the AAS and to characterize its reliability in Asian patients, specifically in Thailand. A structured translation was conducted with approval from the original authors. The Patient Global Assessment of Disease Activity (PGA-DA) was used as an instrument to compare with the Thai version of the AAS. In total, 86 patients with RAE participated in the study. Seventy-six (88%) patients had RAE with chronic spontaneous urticaria. The Thai AAS was found to be a valid and reliable instrument, with high convergent and known-groups validities, excellent internal consistency, and good test-retest reliability. The validity and reliability of the AAS for assessing RAE disease activity in Asian patients have been demonstrated by our study, making it the first to do so. This will help promote the use of the AAS, in clinical trials and practice, in Asia. It will also facilitate the comparison of disease activity in patients with RAE inside and outside Asia in future studies. However, a limitation of this study was its small number of patients.

## 1. Introduction

Recurrent angioedema (RAE) is characterized by the repeated occurrence of nonpitting, skin-colored, or sometimes erythematous swellings of the skin or mucosa, often in the face [1, 2]. Having a valid and reliable patient-reported outcome (PRO) instrument to assess disease activity is important to manage RAE effectively [3]. The Angioedema Activity Score (AAS), which was developed in 2013 in German, is the first PRO measure to assess disease activity in patients with RAE [4]. The EAACI/GA^2^LEN/EDF/WAO guidelines for urticaria recommend the AAS as the standard measurement to assess and monitor the disease activity of patients with RAE [5]. It consists of 5 questions with 4 answer options (scored 0–3) for each item, with a minimum score of 0 and a maximum score of 15 per day. Lower scores and higher scores represent low and high disease activity, respectively. The AAS7, AAS28, and AAS84 are the AASs that describe the disease activity of 7, 28, and 84 consecutive days, respectively [4, 6].

The AAS has been translated and culturally adapted to 80 languages for use in 52 countries [6]. However, it has not yet been formally validated in Asian patients with RAE. As RAE may be different in Asian and non-Asian patients, it is important to validate and characterize the reliability of tools to assess RAE disease activity in Asian patients. Accordingly, this study aimed to generate and validate the Thai version of the AAS and to characterize its reliability in Asian patients, specifically in Thailand.

## 2. Materials and Methods

The Thai version of the Angioedema Activity Score, the AAS, was generated following standard protocols as previously described [7]. In brief, the original AAS was first translated independently by two native Thai-speaking healthcare professionals and reviewed by two additional medical experts. A preliminary consensus Thai version was then backtranslated into German, and this version and the original were compared for discrepancies. Finally, a consensus version was created in collaboration with the authors of the original AAS [4]. All AAS items were easily translated, and the Thai AAS was successfully tested for clarity and comprehension in 10 adult RAE patients.

In total, 86 adult patients with RAE who attended the Allergy Clinic/Urticaria Center of Reference and Excellence [8] of the Department of Dermatology, Siriraj Hospital, were included. Exclusion criteria were a lack of literacy skills and/or other concomitant dermatological or mental diseases. The Thai AAS28 values were compared to results obtained from the Patient Global Assessment of Disease Activity (PGA-DA). The PGA-DA is a self-administered questionnaire that contains one question to assess patient disease activity during a 28-day period. The following 5-point scale is used: 0 = absence, 1 = mild, 2 = moderate, 3 = severe, and 4 = very severe disease activity [9].

At baseline, informed consent was obtained, and the patients were informed about how to complete the Thai AAS and the PGA-DA. After the patients confirmed that they understood how to complete the questionnaires, they were asked to complete the AAS questionnaire for 28 consecutive days by themselves before their second visit. After 4 weeks, the AAS28 was collected and the patients were asked to complete the PGA-DA by themselves. A new AAS28 was then given to each patient to record the disease activity before the third visit. After an additional four weeks, the AAS28 was collected and the PGA-DA was once again completed by the patients.

### 2.1. Validity

- *Convergent validity* measures the relation of the Thai AAS with other standard instruments. The correlation between the Thai AAS and the PGA-DA was determined by Spearman's correlation coefficient. Weak, moderate, and strong correlation was demonstrated as correlation coefficient values of <0.3, 0.3–0.6, and >0.6, respectively [4, 10].
- *Known-group validity* measures the capacity of the AAS to discriminate across groups that are supposed to differ. Using the PGA-DA scores, patients were classified into five groups in this study: (i) “none” (score of 0), (ii) “mild severity” (score of 1), (iii) “moderate severity” (score of 2), (iv) “large severity” (score of 3), and (v) “very large severity” (score of 4). The Kruskal–Wallis test was applied to investigate known-group validity [4, 10].

### 2.2. Reliability

- *Internal consistency* measures the uniformity of AAS items which can indicate whether it is appropriate to calculate a total score. Cronbach's *α* reliability coefficient was used to analyze internal consistency that *α* values of ≥0.9, 0.7 ≤ *α* < 0.9, and 0.6 ≤ *α* < 0.7 were regarded to indicate excellent, good, and acceptable reliability, respectively [4, 11].
- *Test-retest reliability* measures the consistency of the AAS across multiple administrations. Stable patients who have no change in PGA-DA during 4 weeks should demonstrate comparable AASs between two different visits. Intraclass correlation coefficient (ICC) values of <0.40, 0.4–0.75, and >0.75 are evidence of poor, average, and strong reliability, respectively [4, 11].

This study was approved by the Siriraj Institutional Review Board, Faculty of Medicine, Siriraj Hospital (SI 271/2017). SPSS for Windows, Version 18.0 (SPSS Inc., Chicago, IL, USA), was used to analyze data. *p* values ≤0.05 were considered to indicate statistical significance.

## 3. Results

**Figure 1:**
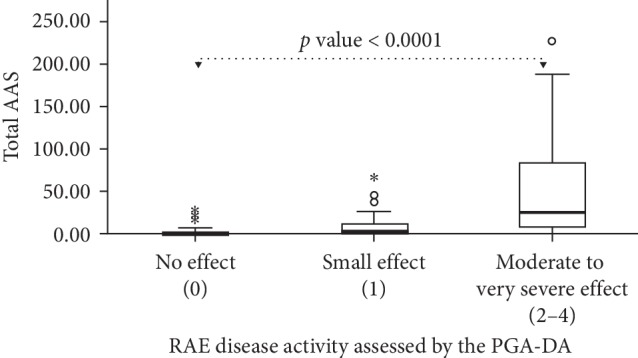
The Thai version of the Angioedema Activity Score (AAS) questionnaire can differentiate patients with recurrent angioedema (RAE) who differ in their disease activity when assessed by the Patient Global Assessment of Disease Activity (PGA-DA). The asterisk and circle indicated the values of ASS scores which were out of 1.5 interquartile range in each group.

Of the 86 patients, 76 (88%) had RAE with concomitant wheals, while the remaining 10 (12%) had RAE only. Demographic data are detailed in Table 1. The convergent validity of the Thai AAS28, i.e., the correlation with the PGA-DA, was strong (Spearman's correlation coefficient *r* = 0.63; *p* < 0.0001). Using the PGA-DA scores, we then classified patients, in terms of their disease activity, into five groups: (i) “absence,” (ii) “mild,” (iii) “moderate,” (iv) “severe,” and (v) “very severe.” This was done to assess the known-group validity of the AAS28, in other words, its ability to discriminate between groups that are different. As determined by the use of the Kruskal–Wallis test, the Thai AAS28 reliably discriminated between patients with low, moderate, and high disease activity (*p* < 0.0001; Figure 1).

Internal consistency measures the uniformity of the items of a PRO measure and indicates whether it is appropriate to calculate a total score. To determine the internal consistency of the Thai AAS, we used Cronbach's *α* coefficient, for which *α* values of ≥0.9, 0.7 ≤ *α* < 0.9, and 0.6 ≤ *α* < 0.7 were regarded as indicating excellent, good, and acceptable consistency, respectively. Cronbach's *α* value of the Thai AAS was 0.97, which indicates excellent internal consistency.

Additionally, we determined the test-retest reliability of the Thai AAS, i.e., its consistency across multiple administrations. Stable patients who have no change in PGA-DA during 4 weeks should demonstrate comparable AASs at two different visits. Intraclass correlation coefficient (ICC) values of <0.40, 0.4–0.75, and >0.75 are evidence of poor, average, and strong reliability, respectively. In the current study, 44 patients had no change in their PGA-DA scores between the two visits. The ICC value of their AAS28 scores was 0.72, which indicates *average* test-retest reliability.

## 4. Discussion

In order to improve the treatment of AE patients, all of the disease severity and impact on HRQoL in each patient need to be recognized by physicians. However, the validated patient-report outcome instrument to determine disease severity in AE patients is very small in number. In 2013, the AAS was established by Weller et al. to be a specific tool to measure disease severity in AE patients [4]. As the AAS is easy to use in clinical practice, its application is recommended by the EAACI/GA^2^LEN/EDF/WAO guidelines for the management of urticaria [5]. The AAS has been designed in such a way that it is convenient to complete and its score is easy to interpret. Moreover, an advantage of the AAS is that it is independent of any previous presentations, making it suitable for application in the event of fluctuating attacks of AE.

Our study demonstrated strong convergent and known-group validities. It illustrated that the higher the AAS is, the more severe the AE is in each patient. Moreover, the known-group validity of the AAS showed a statistically significant difference for each PGA-DA group. This confirms that the AAS is a valid instrument to evaluate disease severity for Asian patients with AE. Moreover, the reproducibility of the AAS was exhibited by excellent internal consistency and good test-retest reliability.

Our study has limitations, namely, the relatively small number of patients and types of RAE investigated and the restricted range of AAS28 values in our patients. As for the latter, we only obtained values between 0 and 228 (mean: 22.0 ± 42.5), even though the full range of the AAS28 is from 0 to 420. Similarly, in a recent study, RAE activity assessed by the AAS7 (the use of the AAS for one week) showed a mean value of 23 even though the AAS7 ranges from 0 to 105 [12]. This is because the maximum values of 105 for the AAS7 and 420 for the AAS28 are “theoretical values.” To have a value of 420 in the AAS28, a patient would have to have severe angioedema attacks 24 hours a day for 28 days straight. Nevertheless, this study found the AAS28 to be a valid and reliable tool for measuring RAE activity in both Western and Asian patients.

## 5. Conclusions

In conclusion, this study is the first to demonstrate the validity and reliability of the AAS28 in Asian patients with RAE. This will help promote the use of the AAS, in clinical trials and practice, in Asia. It will also facilitate the comparison of disease activity in patients with RAE inside and outside Asia in future studies.

## Figures and Tables

**Table 1:** Demographic and clinical characteristics of patients with recurrent angioedema (RAE) (*n* = 86) as well as their response to treatment and disease activity, as assessed by the Thai Angioedema Activity Score 28 (AAS28) and Patient Global Assessment of Disease Activity (PGA-DA).

	Values
Gender, *n* (%)	
Female	69 (80.2)
Male	17 (19.8)

Age, years	
Mean age (range)	38 ± 15 (18–76)

Diagnosis, *n* (%)	
RAE without wheals	10 (11.6)
RAE with wheals	76 (88.0)

Range of scores (full score)	
Thai AAS28	0–228 (420)
PGA-DA	0–3 (4)

Mean scores ± SD	
Thai AAS28	22.0 ± 42.5
PGA-DA	1.1 ± 1.0

Treatment, *n* (%)	
H1-antihistamines	74 (86)
H1-antihistamines + prednisolone	8 (9)
H1-antihistamines + omalizumab	4 (5)

Assessment of disease activity using the PGA-DA questionnaire, *n* (%)	
None	33 (38)
Mild	24 (29)
Moderate	21 (24)
Severe	8 (9)
Very severe	0 (0)

## Data Availability

The data used to support the findings of this study are available from the corresponding author upon request.
